# An Integrated Environment Monitoring System for Underground Coal Mines—Wireless Sensor Network Subsystem with Multi-Parameter Monitoring

**DOI:** 10.3390/s140713149

**Published:** 2014-07-21

**Authors:** Yu Zhang, Wei Yang, Dongsheng Han, Young-Il Kim

**Affiliations:** 1 School of Electronic and Information Engineering, Beijing Jiaotong University, Beijing 100044, China; E-Mail: wyang@bjtu.edu.cn; 2 Faculty of Information Technology, Beijing City University, Beijing 100083, China; 3 School of Electrical & Electronic Engineering, North China Electrical Power University, Baoding 071003, China; E-Mail: 07111039@bjtu.edu.cn; 4 Electronics and Telecommunication Research Institute, Daejeon 305-700, Korea; E-Mail: yikim@etri.re.kr

**Keywords:** multi-parameter monitoring, network management, underground coal mine, wireless sensor network

## Abstract

Environment monitoring is important for the safety of underground coal mine production, and it is also an important application of Wireless Sensor Networks (WSNs). We put forward an integrated environment monitoring system for underground coal mine, which uses the existing Cable Monitoring System (CMS) as the main body and the WSN with multi-parameter monitoring as the supplementary technique. As CMS techniques are mature, this paper mainly focuses on the WSN and the interconnection between the WSN and the CMS. In order to implement the WSN for underground coal mines, two work modes are designed: periodic inspection and interrupt service; the relevant supporting technologies, such as routing mechanism, collision avoidance, data aggregation, interconnection with the CMS, *etc.*, are proposed and analyzed. As WSN nodes are limited in energy supply, calculation and processing power, an integrated network management scheme is designed in four aspects, *i.e.*, topology management, location management, energy management and fault management. Experiments were carried out both in a laboratory and in a real underground coal mine. The test results indicate that the proposed integrated environment monitoring system for underground coal mines is feasible and all designs performed well as expected.

## 1. Introduction

Safety is the top priority of coal mine production and plays an important role in it. In recent years, China has gained great achievements in the field of coal mine production safety, but the situation is still severe. In China, coal mining is mainly carried out underground, where the geological conditions are complicated [1–3]. Along with coal mining at greater depths, the amount of gas emission increases, and thus the risk of coal and gas outbursts grows. Besides, there are other natural factors that can cause disasters in underground mines, such as rock bursts, coal dust explosions and water leaks. With the addition of narrow working space, poor lighting, and many hidden dangers in each coal mining procedure, heavy casualties may occur for slight carelessness. Hence, the hazardous natural conditions and the adverse working conditions make underground coal mines full of numerous risk sources and hidden dangers.

The focus of coal mine production safety is to strengthen monitoring and early warning of possible natural disasters such as gas, fires, pressure, coal dust and floods, since monitoring and controlling, predicting and warning are the precondition and guarantee for the “safety first, prevention crucial” policy, and the key to prevent accidents.

As an important guarantee for coal mine safety production, the Cable Monitoring System (CMS) technology has gone very far, and theoretical problems and the key technology issues involved have been resolved already [1]. Due to the complex working conditions in underground coal mines and the limitations of the CMS, it is difficult for the CMS itself to comprehensively and effectively monitor the environmental parameters which have important impact on the coal mine safety production, such as gas, pressure, coal dust, temperature, wind speed and carbon monoxide (CO) levels. Moreover, the area that is difficult to monitor for the CMS, is generally where the hidden dangers are most prominent, so plenty of hidden dangers are left inevitably, which is a difficult situation for the traditional CMS to overcome. Whereas, by using wireless methods, a wireless monitoring system can effectively, instantly and flexibly monitor the areas that are difficult to monitor for the CMS.

Wireless Sensor Networks (WSNs) are a new kind of *ad hoc* network, which consist of hundreds to thousands of WSN nodes that communicate with each other, and can monitor areas from small to huge [2,4]. In recent years, with the rapid development of mobile communication and high-speed electronic devices, sensors with characteristics of low power consumption, programmability, multi-parameter sensing and wireless communication ability have been put into practical use. With outstanding advantages of ease of configuration, flexibility to shrink or expand the monitoring range, strong fault-tolerance and mobility, WSNs can play an important role in monitoring and analyzing dynamic, hostile and unfamiliar environments. Therefore, based on the WSN technology, a kind of mesh WSN with multi-parameter monitoring for underground coal mines was established, acting as a supplement to the CMS. Since the WSN nodes have multiple interfaces to connect different kinds of sensors, multiple types of environmental parameters can be monitored.

Using WSNs to monitor the environment of coal mines is not a new topic [5–15]. WSN technologies for this application have been investigated before. For instance, strategies for deploying a long distance WSN in an underground coal mine have been put forwarded by Yang [7], Akyildiz has discussed the challenges of using WSNs in underground environments in his theoretical research [12], and Xiao has proposed a multi-path WSN routing protocol for mine security monitoring [14]. Most of these works are theoretical research focusing on certain technology or just focusing on the technologies of the WSN. In this paper, with the aim of practical use, an integrated monitoring system including the WSN and the CMS is constructed.

The rest of this article is organized as follows: Section 2 introduces the overall design of the integrated environment monitoring system. Section 3 introduces the supporting technologies of the WSN. Section 4 discusses network management. Section 5 presents the performance evaluation through experiments. Section 6 concludes this work.

## 2. Overall Design of the Integrated System

### 2.1. System Architecture

**Figure 1.**
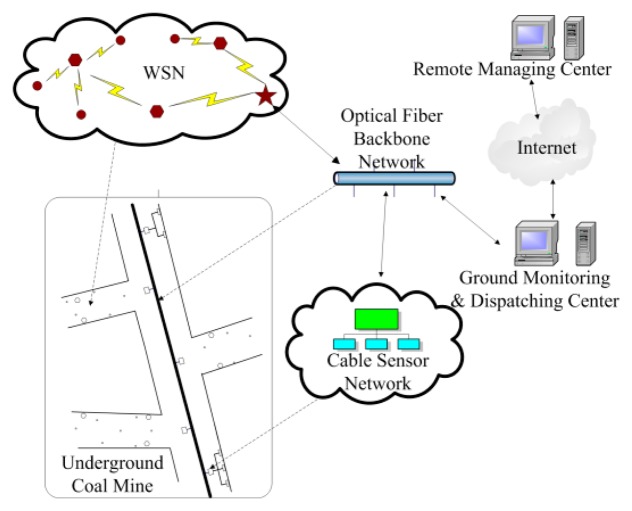
Architecture of the integrated environment monitoring system.

According to the landform, a coal mine can be divided into laneways, mining areas and mined-out areas. The laneways can be further divided into main tunnels and branch tunnels [3]. In the main tunnel and most of the branch tunnels and mining areas, the landform is relatively wide, where the CMS based on an optical fiber backbone network is deployed to monitor the environment. However, the landform in some of the branch tunnels, mining areas, and all of the mined-out areas, is narrow and irregular. It is very inconvenient or even impossible to deploy CMS in these areas. In this case, WSNs are used for environment monitoring because of their flexibility [4,5]. That means the WSN is mainly deployed where is difficult to deploy the CMS. The deployment of the WSN depends on the requirements of comprehensively monitoring the coal mine, which has characteristics of regional dispersion and small relevance with individual WSN. Based on these considerations, an integrated environment monitoring system for underground coal mines is constructed, which can comprehensively monitor the coal mine environment. Figure 1 shows the architecture of the integrated environment monitoring system. As shown in Figure 1, the WSN is mainly deployed in branch tunnels, and communicates with the Ground Monitoring and Dispatching Center (GMDC) via the optical fiber backbone network.

In this architecture, the WSN is composed of three types of nodes: sink nodes, routing nodes and sensor nodes. The sink node establishes a WSN based on the ZigBee protocol [6]. The routing node and the sensor node connect to a sink node to join a WSN. Environmental information about the underground coal mine are collected by the sensor nodes, and are sent to the GMDC through the sink nodes. According to the landform of the coal mine and the environmental monitoring requirements, different WSNs may consist of different kinds of sensors to collect different environment information. The environmental information that the CMS and the WSNs collect are transmitted to the GMDC via the optical fiber backbone network. After receiving the information, the GMDC can know the environmental conditions of the underground coal mine in real time, such as the level of gas and CO, and can rapidly respond to urgent cases. In this case, the monitoring of the environment of the underground coal mine and early warning of dangers could be achieved effectively. In addition, the connection between the GMDC and Internet make it possible for the remote manager to monitor and manage the overall safety conditions of local coal mines.

### 2.2. WSN Nodes Deployment

What is a good network topology? Based on network connectivity and coverage, by adjusting the transmitting power of nodes and selecting some nodes as backbone to deal with data processing and transmission based on certain principles, one can optimize the network topology and extend the lifetime of the whole network.

**Figure 2.**
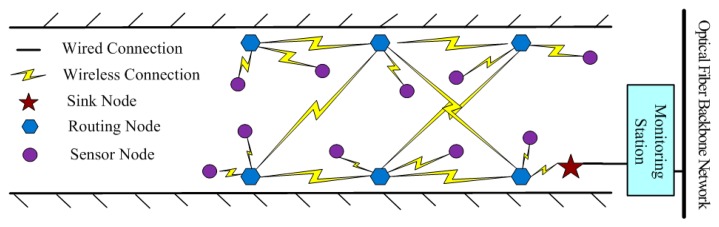
Topology structure of the WSN for underground coal mines.

Due to the harsh environment and complex conditions of wireless communication in underground coal mines, higher requests are put forward on the stability and reliability of the WSN. According to the zonal structure of the coal mine tunnel [7–9], a kind of mesh WSN for underground coal mine is designed based on the ZigBee technology, as shown in Figure 2. In addition, the coal mine is where personnel can come to, so WSN nodes are deployed manually, which is helps optimize and manage the network.

As shown in Figure 2, the WSN for an underground coal mine consists of two layers: the upper backbone network and the lower perception network. The backbone network consists of a sink node and routing nodes; they can communicate with each other directly within their coverage, and mainly gather and forward data. As the gateway between the WSNs and the CMS, the sink node is deployed near the CMS cable monitoring sub-station. According to the zonal structure of the coal mine tunnels, the routing nodes are deployed along the tunnel in two symmetrical lines. In this way, every routing node can communicate with multiple routing nodes directly. If a routing node is broken, communication can still go on by using another routing node for relaying, thus the robustness of wireless communication in WSN is ensured.

The perception network consists of sensor nodes in charge of collecting environment information. They are deployed around the routing nodes and with the consideration of the environmental monitoring needs. Because the sensor node is a Reduced Function Device (RFD), it is designed only to communicate with the nearest routing node as its parent node. Besides, the transmission power of a sensor node is adjusted appropriately by using an adaptive power control mechanism. The transmission power is mainly decided by the Received Signal Strength Indication (RSSI) value between the sensor node and its parent node. By adjusting the transmission power of sensor nodes, not only is the power consumption of the sensor nodes reduced, but also the interference among neighboring nodes is reduced.

### 2.3. Working Modes

As is known, sensor nodes are powered by battery, so their working time is limited by power. In order to prolong the lifetime of sensor nodes, and at the same time ensure the environment of underground coal mine be monitored efficiently and constantly, two working modes are used in WSN, called periodic inspection mode and interrupt service mode.

#### 2.3.1. Periodic Inspection Mode

**Figure 3.**
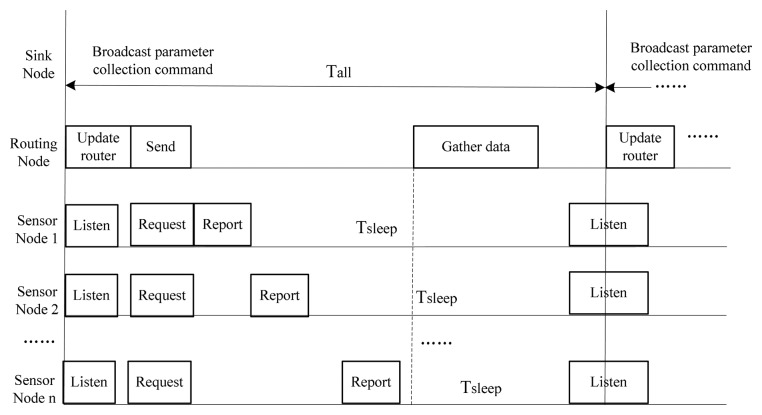
Scheduling of periodic inspection mode.

Based on the deployment of the WSN nodes showed in Figure 2, the scheduling of the periodic inspection mode [11] is used, as described in Figure 3. In each cycle, the sink node starts to broadcast a parameter collection command to the routing node.

After receiving the command, the routing node first forwards it, and then determines whether it has children sensor nodes itself. If it has none, the routing node will send an active status report to the sink node after a random time period; otherwise, it will broadcast the parameter collection command to its children sensor nodes, carrying the network address of the children nodes that it has messages to transmit to, since the sensor node can only communicate with its parent node in an indirect manner. In other words, the sensor node needs to poll its parent node to determine whether it has any messages pending. When sensor node receives the command, it will first determine whether the parent node has data for it. If yes, it requests the data. After this, it collects environmental parameters according to the command, and sends them in its time slot, then goes to sleep to save power. The routing node gathers the parameters received from its children nodes, and then sends them to the sink node in a multi-hop relay way, and finally to the GMDC via the gateway. At this point, a cycle comes to an end.

#### 2.3.2. Interrupt Service Mode

The natural conditions of underground coal mines are so rough that there may be a sudden incident, so it is very important for the safe production in underground coal mines to accurately monitor and provide timely alarms for sudden incidents [7]. Therefore, as the supplement of the periodic inspection mode, the interrupt service mode is designed for the WSN to warn of any abnormal situation. When the sensor node is asleep, the processor and the communication module will be shut down to save power, thus it can neither send nor receive messages, but the sensors will stay active and keep collecting environmental parameters. Once there is an overrun in a certain parameter, the sensor node will be woken up by an interrupt generated by the voltage comparator of the processor. Then, the sensor node marks the parameter as an emergency, and sends it to the GMDC immediately. When GMDC receives the emergency data, it will alarm the corresponding areas about the abnormal situation so as to predict the danger in time.

## 3. Key Supporting Technologies for the WSN

As for the WSN, there are some technologies which are very important for its performance in underground coal mines. These technologies are called key supporting technologies, and mainly include the routing mechanism, collision avoidance, data aggregation, unified parameter gathering, network synchronization and interconnection with the CMS [12,13]. To construct an integrated monitoring system for underground coal mines, all these key supporting technologies have been discussed.

### 3.1. Routing Mechanism

As stated in Section 2.2, the WSN with multi-parameter monitoring for underground coal mines is composed of two layers. For the perception network, the sensor node can only communicate with its parent node, without routing functions. Therefore, the routing mechanism mentioned here is only applied to the backbone network.

Because the WSN nodes are limited in electric power and computing capability, in the first place the routing protocol cannot be complex. Secondly, the underground wireless communication condition is so complex that the routing protocol should be robust enough to guarantee the WSN will work stably and reliably. Thirdly, in the WSN, the sensor node mainly transmits environmental parameters, whose destination is the sink node [14–16]. Based on the above considerations, a kind of routing mechanism with minimum hops, multiple paths and based on link quality indication (LQI) is designed.

In this routing mechanism, every routing node establishes three routes, *i.e.*, parent route, minimum route and backup route, by parsing the parameter collection command which is broadcast periodically. To store the route information, every routing node maintains a routing table, containing items like *hops*, *LQI*, *flag* and *next_hop_address*, as described in Table 1. The destination is the sink node, so the destination address item is omitted.

In order to reduce the power that consumed for transmission, the hops information is carried along in the parameter collection command. The value of hops is zero when the command is first broadcast by the sink node, and adds 1 each time it passes a routing node. After receiving the command, the routing node extracts the hops and LQI information, then the link cost will be obtained based on the definition stated in the ZigBee protocol [6]. The routing node updates the routing table according to the following rules:

(1) Determine whether the command comes from its parent node. If yes, add it into the routing table as the parent route; otherwise, go to 2.
(2) Determine whether the minimum route is empty. If yes, add it as the minimum route; otherwise, compare it with the minimum route. If the link cost is less than that of the minimum route, replace it and replace the previous minimum route with the backup route; otherwise, go to 3.
(3) Compare it with the backup route. If the backup route is empty, or the link cost is less than that of the backup route, add or replace it as the backup route; otherwise, discard directly.

At this point, the routing node finishes updating the routing table in a cycle. When the routing node forwards data, it first chooses the minimum route. If it fails to send, it chooses the parent route then. If it fails to send again, it finally chooses the backup route.

### 3.2. Collision Avoidance Mechanism

According to the ZigBee protocol, WSNs that work at 2.4 GHz frequency have 16 communication channels. In order to reduce the channel interference among WSNs, orthogonal channels are assigned to the WSNs within the interference range. That means the channel of any WSN in the integrated monitoring system is orthogonal to the channels of neighboring WSNs within the interference range.

Within a WSN, all WSN nodes are working over the same channel. If they transmit simultaneously, collisions will happen, and the interference will lead to the failure of data transmission [17]. Collisions happen on both the layers of a WSN: collisions among the routing nodes in the backbone network, and collisions among the sensor nodes with the same parent routing node in the perception network.

To deal with the collisions among routing nodes in the backbone network, a kind of unslotted carrier sense multiple access with collision avoidance (CSMA/CA) algorithm is employed. According to the ZigBee protocol, the unslotted CSMA/CA algorithm means that the WSN node waits for a random time period, namely the avoidance time, before it sends data. After the avoidance time, the node starts to listen to the channel. If the channel is idle, it sends immediately; on the contrary, if the channel is busy, it waits and then tries to access to channel again.

In the WSN, a routing node normally has more than one child sensor node. When these sensor nodes send data to the routing node simultaneously, collisions will happen. For this reason, the time is divided into slots and allocated to each sensor node according to the order in which it joins the routing node. The order is computed through Equation (1):

(1) $$n=A_{n}-A_{\text{parent}}-C_{\text{skip}}(d)*R_{m}$$

where, *n* is the order in its parent node, *A_n_* is the network address of the sensor node assigned by its parent routing node, *A_parent_* is the address of its parent routing node, *C_skip_*(*d*) is the size of the address sub-block being distributed by each parent with depth of *d* to its children routing node, and *Rm* is the maximum number of routing nodes a parent may have as children.

By using this timeslot allocation mechanism, collisions among sensor nodes under the same parent routing node are effectively avoided. It also helps the routing node in gathering environmental information and managing the children sensor nodes.

### 3.3. Data Aggregate Mechanism

In the integrated monitoring system for underground coal mines, a sensor node is equipped with multiple sensors so as to collect different types of environmental parameters. To improve the transmission efficiency, the collected environment data needs to be aggregated before reporting it to the parent routing node. Therefore, a data aggregation mechanism is employed on the sensor nodes. The frame format of the aggregated data is illustrated in Table 2. Here, the *RFD* field shall be set to 0X0F, indicating that this frame is environmental information that the sensor node sends; *Limit* field shall be set to 0X01 if a parameter overruns, otherwise, be 0; *Type* field indicates the type of parameters; *Para* field carries the specific value of parameters.

When it receives environment information from its children sensor nodes, the routing node caches it firstly. After it receives the data from all the children nodes, it aggregates it and then sends it to the sink node. The frame format of the packaged data is illustrated in Table 3. Here, the *ROUTER* field shall be set to 0X0E, indicating that this frame is environmental information that the routing node is sendsing; *Num* field indicates the number of sensor nodes in this frame; *Addr* field means the network address of each sensor node; fields of *Type and Para* have the same meaning with the corresponding items in Table 2. Because the overrun parameter is sent directly to the sink node by the sensor node, *Limit* field is not included in the frame.

In the environmental information delivery process, data aggregation is adopted by the sensor node and the routing node, which decreases the amount of data, reduces data collisions, saves power, and extends the lifetime of the whole network. Meanwhile, for the overrun parameters, the sensor node directly sends the to the sink node without aggregation, realizing urgent transmission of the emergency data.

### 3.4. Unified Parameter Gathering Mechanism

As mentioned above, there are many kinds of sensors in the WSN in charge of monitoring the environment comprehensively. How to gather and recognize the different sensing data efficiently? A kind of unified parameter gathering mechanism is presented. For example, we need to collect the following eight kinds of parameters: gas, CO, coal dust, smoke, temperature, humidity, wind speed and pressure, 1-byte *Type* field in the frame as shown in Table 3 is used for expressing the type of parameters. Table 4 shows the relationship between Type-value and the corresponding parameter type, where 0x00 means that he sensor nodes are allowed to go to sleep.

As shown in Table 4, each bit of the *Type* Byte represents a kind of environmental parameter. If a kind of parameter is collected, the corresponding bit is set 1, otherwise, set 0. After receiving a parameter collection command, the sensor node starts to collect parameters according to the *Type* value in the command. If the requirement of the sensor types is more than the types of sensors with which that the sensor node is equipped, then the *Type* value should be reset according to the actual sensor types. For instance, a sensor node is equipped with two sensors: a gas sensor and a CO sensor. When it receives a command of Type value 0x07, which means collecting three gas parameters, CO and coal dust, then the *Type* value should be set 0x03. In this way, various environment parameters are gathered uniformly although different sensor nodes may be equipped with different sensors.

### 3.5. Network Synchronization Mechanism

The mesh WSN with multi-parameter monitoring for underground coal mines mainly works in the periodic inspection mode, but the accuracy of the crystal oscillators of the WSN nodes is generally low, which means their clocks are not accurate, so it is hard to keep WSN nodes working synchronously. In this case, a relative synchronization mechanism is proposed. In this mechanism, the sink node, which is the coordinator of the WSN, is selected as the basic benchmark node of the WSN. The other nodes select their own parent node as their benchmark node. The designed network synchronization means relative synchronization on working and sleeping, rather than absolute synchronization on a physical clock [3]. As the coordinator, the sink node is selected as the benchmark node of the whole WSN, and other WSN nodes select their own parent nodes as the benchmark node.

As designed, the sink node periodically broadcasts the parameter collection command. The command frame is also the synchronous frame in the proposed synchronous mechanism. When a WSN node receives a synchronous frame, it will check whether the synchronous frame comes from its parent. If not, it discards it directly; otherwise, it compares the sequence number of the frame with that of the last one. If they are identical, it discards the frame directly; otherwise, it records the sequence number, and regards that moment as the beginning of a new cycle.

In addition, in order to reduce the impact of clock skew, the sensor node listens to the channel both at the beginning and the end of each cycle until it receives a synchronous frame, rather than only at the beginning. In this way, the WSN nodes are capable of working synchronously without accurate clocks, ensuring the reliable delivery of environmental information.

### 3.6. Interconnection between WSN and CMS

In order to transmit the environmental information that the WSN collects to the GMDC stably, the WSN is designed to interconnect with the optical fiber backbone network via the traditional CMS, and the WSN interconnects to the CMS through the sensor node by using a Modbus protocol [18]. In addition, the sink node in the WSN connects to the CMS via a serial port. The sink node has a RS232 interface, while the CMS has a RS485 interface, so RS232/RS485 conversion equipment is used.

The Modbus protocol is a common language used by electronic controllers. It defines a message structure that controllers will recognize and use, regardless of the type of networks over which they communicate. It describes the process a controller uses to request access to another device, how it will respond to requests from the other devices, and how errors will be detected and reported.

The Modbus protocol is based on a master/slave or request/reply architecture. For our integrated environment monitoring system, the master is the cable monitoring station—it requests a message. The slave is the WSN, it takes action and responds to the master. In a Modbus network, there is one master and up to 247 slaves, each with a unique slave address from 1 to 247. For saving address, only the sink node is assigned an address for the WSN. The sink node gathers the environment information of the whole WSN, hence the information can be obtained only through the sink node.

The Modbus protocol supports two transmission modes: ASCII and Remote Terminal Unit (RTU), the RTU mode is used in the interconnection. The RTU format uses binary coding, each byte in a message contains two 4-bit hexadecimal characters, and each byte consists of a start bit, 8 data bits, a bit for even/odd parity, and a stop bit. The main advantage of this mode is that its higher character density allows better data throughput than ASCII for the same baud rate.

## 4. Management for the WSN

In the mesh WSN with multi-parameter monitoring for underground coal mines, the WSN nodes are limited in power quantity, calculation and processing power. Moreover, they usually work without manual intervention, so some WSN nodes may fail. To ensure the stability of the WSN operation and the reliability of the data transmission, it is necessary to monitor the network health in real time, diagnose the network anomaly in a timely way, find rational solutions automatically or manually, and maintain the normal operation of the WSN [19–21]. Therefore, an integrated network management scheme is designed based on four aspects, *i.e.*, topology management, location management, energy management and fault management.

### 4.1. Topology Management

Topology discovery means acquiring the topology structure of the WSN and the physical links between WSN nodes [19]. It is the main job of topology management. A simple but effective kind of WSN topology management is proposed, whereby the mesh network topology can be obtained according to the neighbor table and the relationship between WSN nodes.

After the sink node establishes a ZigBee network, the routing node and the sensor nodes join it, and register their network address, physical address and their parent nodes' network address in the sink node. When the sink node receives this information, the tree topology can be established through the relationship between parent and child. After the WSN works steadily, the sink node knows all the routing nodes' neighbor tables by broadcasting a neighbor table request command, and the mesh topology can be established. During the operation, when nodes' status or links' status changes, the change will be reported to the sink node. According to this information, the sink node dynamically updates the topology structure, reflecting the operation situation of the WSN in real time.

### 4.2. Location Management

It is important to match the parameters that the WSN collects with their position, which benefits from analyzing environment information within specific areas and helps warn abnormal areas accurately [20]. Therefore, a kind of regional location mechanism is designed to estimate the rough location of WSN nodes in underground coal mine. For the sink node and the routing nodes, their number is not very large and their deployment is regular, so their location coordinates are configured manually. While the sensor nodes are close to the routing nodes, they are located within the parent's communication range. Then, according to the RSSI value between them, more precise locations can be obtained, reducing the complexity of network configuration.

When a WSN node lacks power or breaks down, its exact location in the underground coal mine needs to be determined, but the above location mechanism fails. Therefore, a unique identification (ID) is assigned for each WSN node, and is posted on the node. Meanwhile, their corresponding relationships are stored in the database. When the user locates a WSN node, he can rapidly determine its location according to its location coordinates, and then find the specific node based on the ID information.

### 4.3. Energy Management

The WSN nodes mainly use batteries to provide power, so their power is limited. Although some energy conservation measures are adopted, for instance, using a fixed channel allocation scheme, letting the sensor nodes work and sleep alternately and aggregating the environmental parameters, the WSN nodes still may fail for lack of power. Therefore, it is necessary to monitor the remaining power of each WSN node in real time and warn of the remaining power in a timely way [21].

After the WSN nodes join the network, they will report their remaining power to the GMDC. When the user wants to know the current remaining power, it will broadcast a power request command. After receiving the command, the WSN nodes will respond as soon as possible. In addition, based on the characteristic of processor, a function called low voltage detection is designed. When the processor detects that its voltage is lower than a given threshold (set in advance), it will generate a low voltage interruption to the GMDC. According to the warning, the user knows that a WSN node lacks power, and a technician can locate the node based on the location scheme introduced in Section 4.2 and change the batteries, ensuring the normal operation of the WSN.

### 4.4. Fault Management

Once a WSN is deployed, it works for a long time, during which a node or a link may break down, affecting the normal operation of the WSN. Although the WSNs based on the ZigBee protocol have a self-recovery function, it doesn't work in the case of hardware faults [22]. Hence, a fault management scheme is necessary to diagnose the network faults and repair them in time, so as to ensure the normal operation of the WSN.

As mentioned in Section 2.2, the mesh WSN for an underground coal mine is composed of two layers. In the backbone network, the routing nodes communicate with the GMDC via the sink node; in the perception network, the sensor nodes communicate with the sink node via the routing nodes. Based on this hierarchical structure, a hierarchy and cluster machinery is adopted to manage the WSN. The WSN is divided into two layers to manage it. In the backbone network, the sink node is the Cluster Head (CH), the routing node is the Cluster Member (CM); in the perception network, the routing node is the CH, while the sensor node is the CM. The CH is mainly responsible for managing the CMs, reporting the environmental information and the network status within its cluster to the upper network, and forwarding the commands from the upper network. By using this hierarchy and cluster machinery, management tasks are decentralized, realizing effective management for the WSN.

In the process of the WSN gathering the environmental information periodically, the information gathered by CMs must be reported via the CH to the upper network. Therefore, in each cycle, the CH knows which CMs have reported and which not. If the CH does not receive information from a CM twice, it considers that the CM may be broken. In order to determine whether the CM is really broken, the CH sends a state request command to the CM, and starts a Tcm timer. If the CH receives a reply from the CM within Tcm, it means the CM is normal; otherwise, the CH considers that the CM is really broken, and reports to the GMDC to inform the user of the failure node.

Because a sensor node can only communicate with its parent routing node, if the parent node fails, the sensor node is isolated from the sink node even if it is normal. Based on this, it is designed that if a sensor node does not receive an acknowledgement frame (ACK) from its parent node N (e.g., N = 3) times, it sets the transmitting power to maximum, and then tries to communicate with its parent. If it still fails, it considers the parent has failed, and scans the channel to rejoin the network.

According to the network status information that the GMDC receives, the failed nodes can be known and the causes can be diagnosed. Based on the location scheme introduced in Section 4.2, it is easy to locate the corresponding failure node and then remove the fault. After failure recovery, the node should be put back in its place, and its physical address should stay the same, so that the node's network address and neighbor table can be the same as before after it joins the network again, guaranteeing the stability of the network topology.

## 5. Experiment and Performance

In order to estimate the performances of the proposed schemes, including WSN technologies, WSN management and the interconnection between the WSN and the cable monitoring system, the corresponding tests are carried out. Firstly, experimental WSN systems are established in the laboratory environment to test the performance of the WSN technologies and WSN management schemes. Then, to test data transmission reliability of the designed WSN when it works in a real complex underground coal mine environment, tests were carried out in an actual underground mine. Finally, tests were performed using an existing CMS to estimate the interconnection scheme between the WSN and the CMS.

### 5.1. Laboratory Experiments

A test was performed in a laboratory corridor which is about 90 m long, 2 m wide and 2.5 m high. The corridor simulates the underground coal mine laneway, while the rooms and the stairs along the corridor simulate the mining areas and the mined-out areas. As the landform of the laboratory is similar to the landform of an underground coal mine [23], tests were first carried out in the laboratory to examine the performance of the proposed WSN technologies and WSN management schemes.

**Figure 4.**
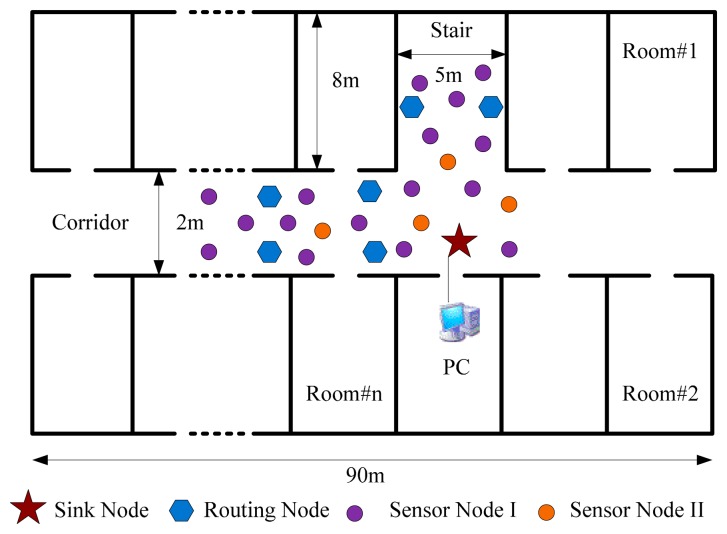
Testing in the laboratory environment.

When the transmission power of the WSN nodes was 0 dbm, the communication distance was about 32 m [24,25]. A WSN was established with one sink node, six routing nodes and 20 sensor nodes, and the network depth was four. The routing nodes were placed along the corridor in two symmetrical lines with an interval of 15 m, as depicted in Figure 2, and their transmission powers were all set to 0 dbm. The sensor nodes were scattered around the routing nodes randomly. There were two types: (I) one equipped with a MC112 gas sensor, a LM35 temperature sensor and a HM1 humidity sensor, which numbered 16; (II) the other equipped with a random number generator, with threshold value of 0XC0 and number of four, that means it overruns if the value is greater than 0XC0. The sink node is connected with a PC via a RS232 interface. The PC simulated the GMDC, it could display environmental parameters and information about the network state received from the WSN on a tool called serial debug terminal, and it could also publish commands to the WSN via the tool. The laboratory environment is shown in Figure 4. The working period was set to 5 min, and the working state of the network was recorded continuously for 3 h.

**Figure 5.**
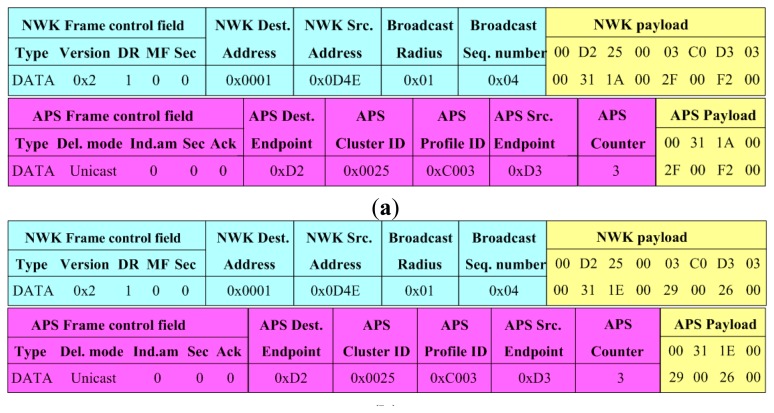

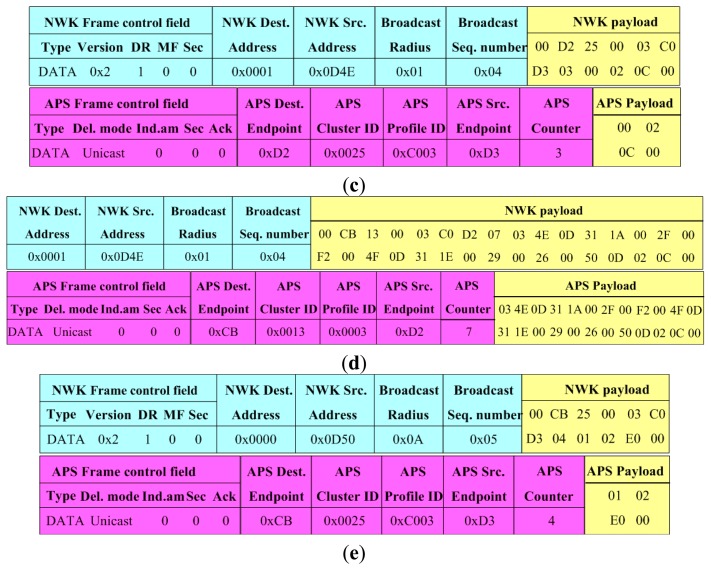
Data frames during communication between a routing node and its three children sensor nodes. (**a**) Sensor node 0X0D4E sending normal parameters of gas, temperature and humidity to 0X0001; (**b**) Sensor node 0X0D4F sending normal parameters of gas, temperature and humidity to 0X0001; (**c**) Sensor node 0X0D50 sending normal parameters of random number to 0X0001; (**d**) Router 0X0001 gathering parameters from (a–c); (**e**) 0XE0 is larger than 0XC0, so 0X0D50 sends it to the sink node directly with Limit of 1As can be known from Figure 5, the WSN ran well both in periodic inspection mode and in interrupt service mode. The sensor nodes could correctly collect environmental parameters according to the command and aggregate them, and they could also report the overrun parameter to the sink node instantly. The routing nodes could correctly aggregate the parameters received from different types of sensor nodes.

Through a tool called Packet Sniffer running on the PC, some data frames were captured during communication. Figure 5 shows part of data frames about the routing node 0X0001 communicating with its three children sensor nodes 0X0D4E, 0X0D4F and 0X0D50, where, the sensor nodes 0X0D4E, 0X0D4F belonged to type (I), while the sensor node 0X0D50 belonged to type (II). By analysis of the data frames, information such as frame type, source address, destination address and application layer data could be parsed easily.

**Figure 6.**
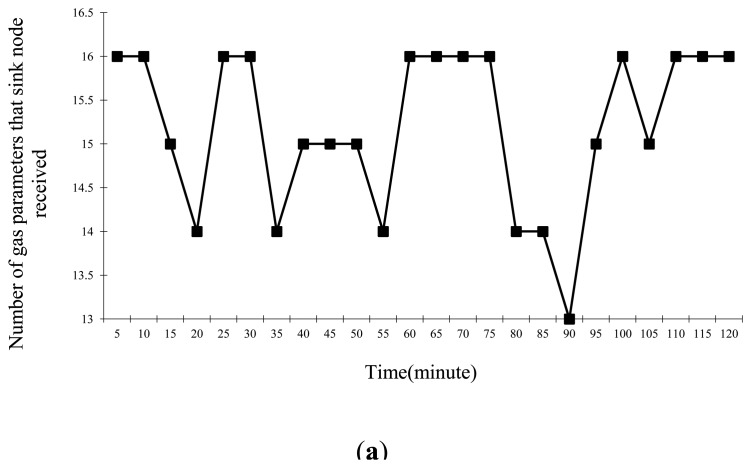

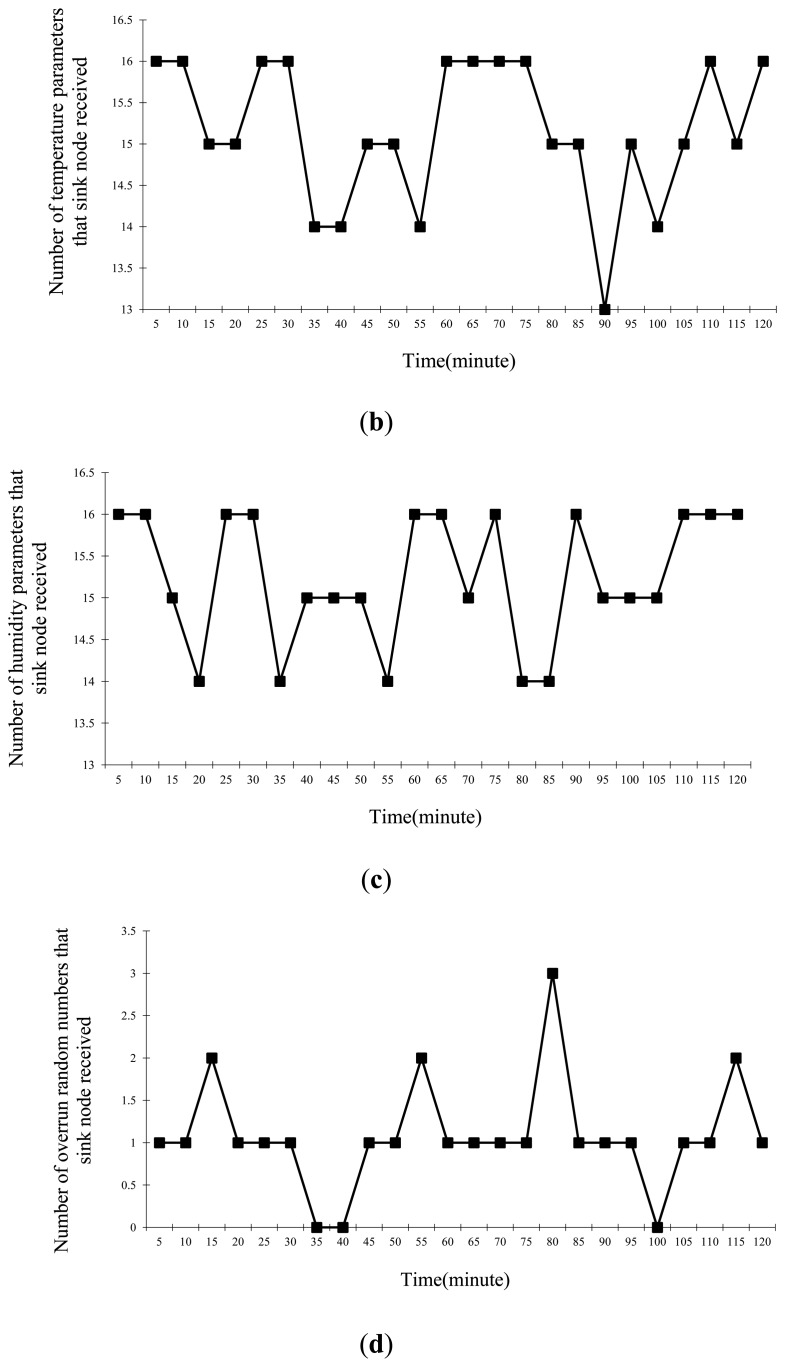
Statistics of each parameter in the laboratory environment. (**a**) Statistics of gas parameters; (**b**) Statistics of temperature parameters; (**c**) Statistics of humidity parameters; (**d**) Statistics of random number.

After the test, by calculating the number of the sensor nodes that sent each type of environmental parameter and the overrun parameter to the sink node in each period, the stability of the WSN was obtained, the statistics areshown in Figure 6.

As plotted in this figure, we can see that the sink node received parameters from most of sensor nodes every 5 min, meaning the periodic inspection function is stable; Meanwhile, it received some parameters from a few sensor nodes, their Limit field was 0X01 by parsing these frames, that is, they were overrun parameters, so the interrupt service function is stable. We note that the sink node did not receive parameters from all sensor nodes in some periods. Nevertheless, the number was relatively small so that we have reason to consider that the WSN works stably.

Finally, the performance on the management scheme was tested. After the establishment of the WSN, the serial debug terminal received information on network address, physical address, coordinate location, remaining power, and their parent's network address from all WSN nodes. According to the information, the network tree topology, the rough location and the remaining power of each WSN node could be obtained. Therefore, the functions of topology management and energy management are working stably. In order to test the fault management, a WSN node's electricity power was shut off manually, then the serial debug terminal received a message that a WSN node failed after several periods. By parsing the network address, the failed node was identified. Based on the failed node's coordinates and ID posted on it, it could be quickly located in the corridor. Thereby, the functions of location management and fault management are both working well.

Based on the above experiments, we conclude that the designed mesh WSN with multi-parameter monitoring for underground coal mine is easy to establish and works well. In both periodic inspection mode and interrupt service mode, the WSN can correctly collect environmental information, aggregate it, and provide early warning of overrun parameters. The proposed network management scheme is easy to implement, it can correctly reflect the network topology, diagnose the network faults in a timely way and repair them, thus ensuring the stable operation of the WSN.

### 5.2. Experiment in Real Underground Coal Mine

**Figure 7.**
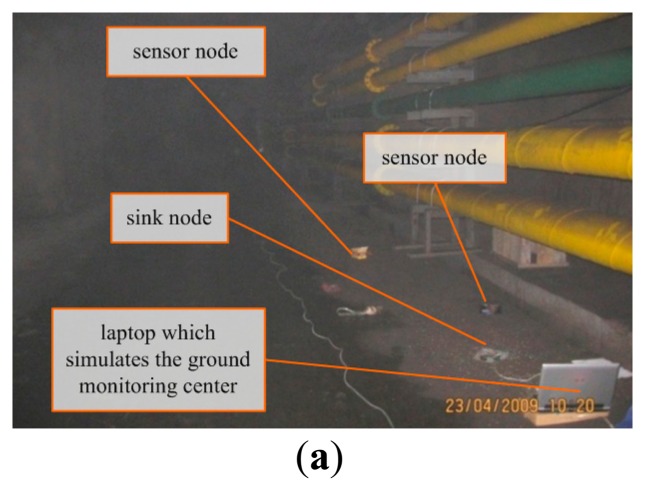

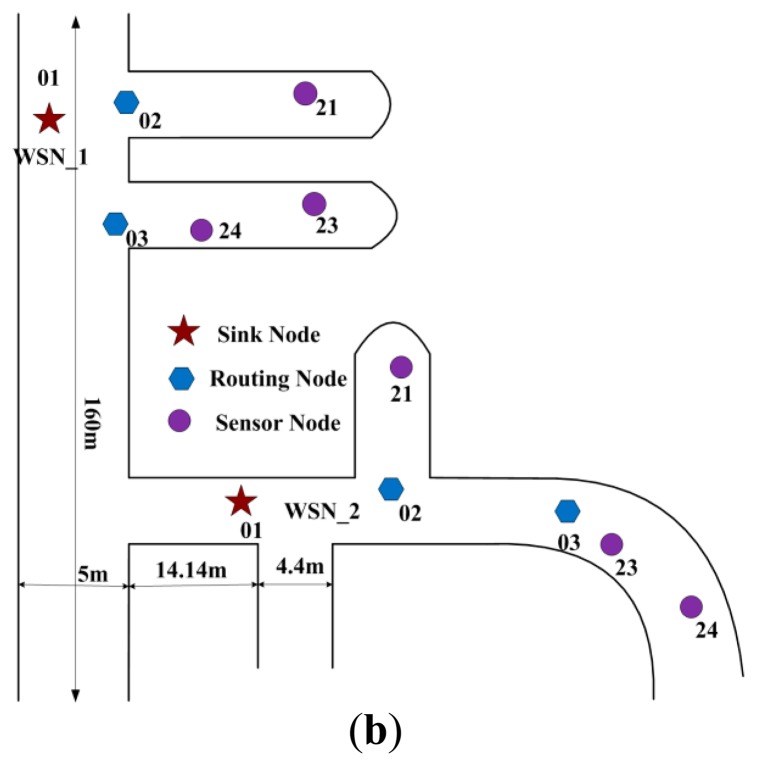
(**a**) Testing scenario in an underground coal mine; (**b**) Deployment of WSNs.

The field test was carried out in the Shangwan coal mine, located in Erdos (Inner Mongolia, China). The test environment is shown in Figure 7, where there is a main tunnel with a depth of about 160 m, width of 5 m and height of 4 m, and there are multiple branch tunnels along the main tunnel. Due to the underground coal mine management rules, the scale of our testing network is restricted. In this case, only two mesh WSNs were deployed: WSN_1 and WSN_2. Each WSN contains a sink node, two routing nodes and three sensor nodes. Each sensor node has three sensors: MC112, LM35 and HM1, thus is capable of collecting three types of parameters of gas, temperature and humidity, as shown in Figure 3. Figure 7b depicts the testing environment and the deployment of the WSNs.

In the field test, the parameters are almost the same as the parameters of the laboratory tests, except the working period is 3 min not 5 min. To avoid influencing the normal production of the coal mine, the field test only lasted for 1 h and mainly tested the data transmission reliability.

The test results are recorded in Table 5, where, the Number of periods means the times that the sensor nodes transmitting data frames to the sink node while the Number of received data means the data frames the sink node had successfully received. As shown in Table 5, the WSNs are working reliably.

### 5.3. Experiment on Interconnection between the WSN and the CMS

The interconnection testing was carried out by interconnecting the designed WSN with the KJ95N cable monitoring system which was developed by the Changzhou Institute of Automation which belongs to the China Coal Research Institute (Beijing, China). The sink node in the WSN was connected to the KJF16B monitoring station via a RS232/RS485 conversion device; and the KJF16B was connected to the KJ95N monitoring and dispatch center via a RS485 interface. KJF16B is an embedded monitoring station based on an ARM7 core processor. The environmental information of the underground coal mine is transmitted to the GMDC through the KJF16B, and the information can be displayed on its LCD. During the test, the working parameters of KJF16B were configured by the KJ95N. Three types of environmental parameters were added, including gas, temperature, and smoke. The baud rate of serial data transfer was set to 1,200 bits per second (bps).

**Figure 8.**
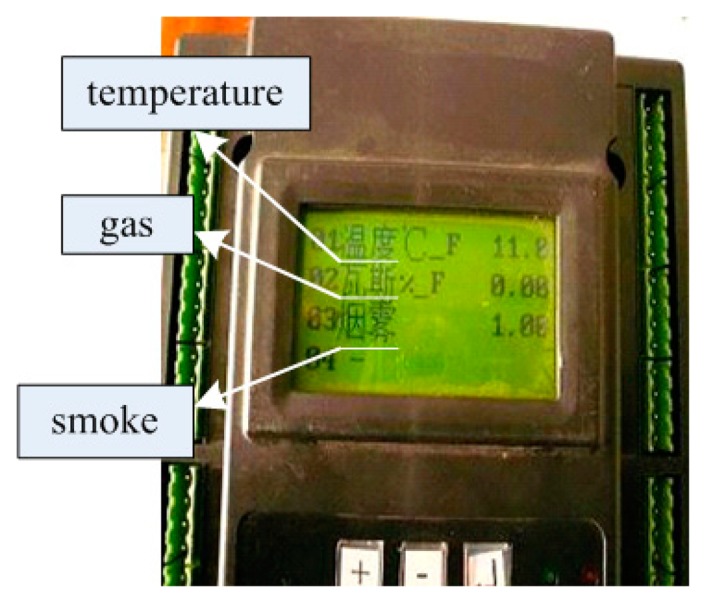
Test results displayed on the KJF16B.

The test data is as shown in Figure 8. The data from the WSNs is displayed on the LCD of the KJF16B monitoring station, including temperature, gas and smoke. The test results prove that the WSNs and cable monitoring system can be well connected by using the proposed interconnection scheme.

## 6. Conclusions

Environment monitoring is important for the safety of coal mine production. As the traditional cable monitoring system (CMS) cannot collect all the required environmental parameters and is difficult to deploy in complex areas such as the mining areas and the mined-out areas of mines, WSN is investigated to monitor the environment in underground coal mines. An integrated environment monitoring system for underground coal mines using CMS as the backbone network and the WSN as the supplementary network is proposed. In detail, the following aspects were studied:

(1) The architecture of the system was designed; the deployment scheme and the working modes of WSN were put forward in detail.
(2) To ensure the stable and effective operation of the WSN, a set of key supporting technologies were discussed, including a routing mechanism with minimum hops and multiple paths based on LQI, a collision avoidance mechanism in combination of CSMA/CA and time division multiple access, a data aggregation strategy, a unified parameter gathering scheme and a relative synchronization scheme to make the network work synchronously. Finally, the method of interconnecting the WSN and the CMS was proposed so as to transmit the underground data of WSN to the GMDC.
(3) As the WSN nodes have limited energy, calculation and processing power, an integrated network management scheme was designed in four aspects, *i.e.*, topology management, location management, energy management and fault management, so as to monitor the network continuously and ensure the stable operation of the WSN.

Experiments were performed both in the laboratory and in an underground coal mine, to test the performance of the designed WSN, the proposed management scheme and the interconnection method between the WSN and CMS. The results showed that the WSN and the management scheme worked well as expected, and the interconnection between the WSN and the CMS could ensure the reliable transmission of monitoring data from the WSN to the GMDC.

The technologies used in the proposed system are all designed based on their suitability for the underground coal mine surveillance application, therefore some of them may not be so advanced in theory, but they were selected because they could make the system working smoothly as a whole. The proposed system has some good features such as universality and expansibility, and we anticipate it can be used for environment monitoring for other relevant fields.

## Figures and Tables

**Table 1.** Routing table.

Item	Size (Byte)	Description
*hops*	1	number of routing nodes from itself to the sink node
*LQI*	1	LQI of the last communication
*flag*	1	distinguish among parent route, minimum route and backup route
*next_hop_address*	2	network address of the next hop to the sink node

**Table 2.** Frame format on sensor node.

1 Byte	1 Byte	1 Byte	Variable
*RFD(0X0F)*	*Limit*	*Type*	*Para*

**Table 3.** Frame format on routing node.

1 Byte	1 Byte	2 Byte	1 Byte	Variable	……	2 Byte	1 Byte	Variable
*ROUTER (0X0E)*	*Num*	*Addr 1*	*Type 1*	*Para 1*	……	*Addr n*	*Type n*	*Para n*

**Table 4.** Relationship between type-value and parameter type.

Pressure	Wind Speed	Humidity	Temperature	Smoke	Coal Dust	CO	Gas	Type Value
0	0	0	0	0	0	0	0	0x00
0	0	0	0	0	0	0	1	0x01
0	0	0	0	0	0	1	0	0x02
0	0	0	0	0	0	1	1	0x03
0	0	0	0	0	1	0	0	0x04
0	0	0	0	0	1	0	1	0x05
-	-	-	-	-	-	-	-	-
1	1	1	1	1	1	1	1	0xFF

**Table 5.** Transmission results of field test. (**a**) Statistics of WSN_1; (**b**) Statistics of WSN_2.

**ID of Sensor Node**	**Number of Periods**	**Number of Received Data**
21	12	11
23	12	10
24	12	10

**(a)**		

**ID of Sensor Node**	**Number of Periods**	**Number of Received Data**

21	18	17
23	18	17
24	18	16

**(b)**		
